# Influence of Maternal Glycemia on Intrauterine Fetal Adiposity Distribution after a Normal Oral Glucose Tolerance Test at 28 Weeks Gestation

**DOI:** 10.1155/2011/951203

**Published:** 2011-10-27

**Authors:** Nadine Farah, Jennifer Hogan, Vicky O'Dwyer, Bernard Stuart, Mairead Kennelly, Michael J. Turner

**Affiliations:** UCD Centre for Human Reproduction, Coombe Women and Infants University Hospital, Dublin 8, Ireland

## Abstract

*Objective*. To examine the relationship between maternal glucose levels and intrauterine fetal adiposity distribution in women with a normal oral glucose tolerance test (OGTT) at 28 weeks gestation. *Study Design*. We recruited 231 women with a singleton pregnancy. At 28 and 37 weeks gestation, sonographic measurements of fetal body composition were performed. Multiple regression analysis was used to study the influence of different maternal variables on fetal adiposity distribution. *Results*. Maternal glucose levels correlated with the fetal abdominal subcutaneous tissue measurements (*r* = 0.2; *P* = 0.014) and with birth weight (*r* = 0.1; *P* = 0.04). Maternal glucose levels did not correlate with the fetal mid-thigh muscle thickness and mid-thigh subcutaneous tissue measurements. *Conclusion*. We found that in nondiabetic women maternal glucose levels not only influence fetal adiposity and birth weight, but also influence the distribution of fetal adiposity. This supports previous evidence that maternal glycemia is a key determinant of intrauterine fetal programming.

## 1. Introduction

Gestational diabetes mellitus (GDM) has been associated with increased birth weight and fetal macrosomia [1]. Increased birth weight is associated with increases in the rates of instrumental vaginal delivery, cesarean section, perineal trauma, and postpartum haemorrhage [2, 3]. Fetal macrosomia increases the risk of shoulder dystocia, brachial plexus injury, intrapartum death, admissions to the neonatal unit, and newborn metabolic problems, including hypoglycemia [4]. Large babies are also predisposed to childhood obesity and to increased morbidity in later life such as hypertension, insulin resistance, and diabetes mellitus [5, 6]. 

Recently, the Hyperglycaemia and Adverse Pregnancy Outcomes (HAPO) study found that in 25,505 women with a normal oral glucose tolerance test (OGTT) at 24–32 weeks gestation, maternal glycemia is associated with a birth weight >90th centile and with neonatal adiposity measured using skinfold callipers postnatally [7, 8]. These findings provided further support for the importance of the intrauterine environment in programming lifelong health and raise the possibility that optimizing maternal glycemia may improve pregnancy outcomes [5].

Over the past 20 years, several investigators have examined the role of fetal soft-tissue measurements in the evaluation of fetal growth abnormalities [9]. Fetal adiposity approximately increases tenfold between 19 and 40 weeks gestation [10]. As a result of accelerated rate of growth in late gestation, measurements of the fetal adiposity, as well as estimates of fetal weight, may potentially be useful in the evaluation of fetal growth abnormalities [9]. The purpose of this study was to examine the relationship between maternal glucose levels at 28 weeks gestation and intrauterine fetal adiposity distribution in women with a normal OGTT.

## 2. Materials and Methods

We conducted a longitudinal prospective observational study in a large university teaching hospital. To minimize confounding variables, the study was confined to white European women with a singleton pregnancy. Women with chronic medical problems that predispose to abnormal intrauterine growth were excluded as well as cases of known congenital fetal malformations.

Women were enrolled at their convenience between July 2008 and December 2009 when they presented for an OGTT. It is hospital practice to selectively screen for GDM at 28 weeks gestation, based on risk factors [11]. A normal glucose response in pregnancy was defined as a fasting value of <5.3 mmol/L, a 1-hour postprandial value of <10.0 mmol/L, a 2-hour postprandial value of <8.6 mmol/L and a 3-hour postprandial value of <7.8 mmol/L after a 3-hour 100 g OGTT [12]. 

All the women enrolled had an early pregnancy ultrasound scan previously to confirm gestational age. In early pregnancy weight and height had been measured digitally in a standardized way, and the body mass index (BMI) calculated [13]. At recruitment, maternal weight was again measured and sonographic evaluation of fetal body composition was carried out by one operator (NF) using a transabdominal curved array transducer on an ALOKA prosound *α*7. The fetal head circumference (HC), biparietal diameter (BPD), abdominal circumference (AC), and femur length (FL) were measured. 

The fetal abdominal subcutaneous tissue was measured on the anterior abdominal wall in millimetres anterior to the margins of the ribs, using magnification at the level of the AC [14]. The fetal mid-thigh muscle thickness was measured at the level of the femur diaphysis as the distance from the outer border of the femur to the inner edge of the subcutaneous layer. The fetal mid-thigh subcutaneous tissue was calculated by subtracting the distance from the outer border of the femur to the outer border of the subcutaneous layer from the thigh muscle. At 37 weeks gestation, maternal weight and fetal body composition were measured again by same operator (NF). Birth weight was measured at delivery. The clinical outcomes of the pregnancy were obtained from the obstetric records. 

Statistical analysis was performed using SPSS version 15.0 (SPSS Inc. Chicago, Ill, USA). Relevant descriptive statistics (mean, standard deviation and percentages) were obtained for the study population. All the variables were checked for normality using the Kolmogorov-Smirnov test. The relationship of fetal body composition measurements and birth weight to a range of possible explanatory variables was investigated, in the first instance, by use of Pearson correlation coefficients. Explanatory variables identified as significant in this bivariate analysis were subsequently entered into a multiple regression model. Trajectories were tested using repeated measures ANOVA with the predictors identified in the regression analysis.

To determine whether there is a sex difference in the fetal parameters measured the independent Student's *t*-test was used. The 5% level of significance was used throughout. Written informed consent was obtained and the study was approved by the Hospital Research and Ethics Committee.

 In a sample of 25 women, two measurements of the fetal abdominal subcutaneous tissue, mid-thigh muscle thickness and mid-thigh subcutaneous tissue were carried out 5 minutes apart. The test-retest reliability, based on Bland-Altman analysis, for the fetal abdominal subcutaneous tissue, mid-thigh muscle thickness and mid-thigh subcutaneous tissue measurements yielded limits of agreement of (−0.64, 0.74), (−1.03, 1.08), and (−0.77, 0.65), respectively, with coefficients of repeatability of 10.0%, 7.5%, and 11.5% respectively [15].

## 3. Results

In total, 320 women were initially enrolled into the study of which 61 women did not attend for a second visit assessment at 37 weeks gestation. Of those women that did not to attend, 20 women delivered before their second assessment visit at 37 weeks gestation. In the group of women that attended for their second assessment, 28 women were excluded from the study because of an abnormal OGTT. 

The mean age of the final study population (*n* = 231) was 30.6 ± 4.8 years with 41.1% (*n* = 95) primigravidas. The mean gestation at the first antenatal visit was 12.3 ± 1.6 weeks and the mean early pregnancy BMI was 28.2 ± 6.0 kg/m^2^. Of the women studied, 36.4% (*n* = 84) had a normal BMI, 28.6% (*n* = 66) were overweight, and 34.7% (*n* = 81) were obese according to the WHO classification. The mean birth weight was 3.7 (±0.5) kg and 3.9% of the babies had a birth weight ≥4.5 kg. The characteristics and outcomes of the study population are shown in Table 1. 

At the normal OGTT, the maternal fasting and one-hour postprandial glucose levels correlated positively with maternal early pregnancy BMI (*r* = 0.2; *P* < 0.001). The fasting and one-hour postprandial glucose levels did not correlate with maternal age, parity, height, and gestational weight gain (GWG) between booking and 28 weeks gestation. 

The two-hour postprandial glucose level correlated with maternal age and early pregnancy BMI (*r* = 0.2 and 0.1; *P* = 0.016 and 0.04, resp.). The three-hour postprandial glucose level correlated with maternal height and GWG between the booking visit and 28 weeks gestation (*r* = 0.2 and 0.1; *P* = 0.016 and 0.046, resp.).

There were no differences in the mean maternal age, height, early pregnancy weight, and BMI between women with a male fetus and women with a female fetus. There were also no differences in maternal gestational weight gain between booking and recruitment and between recruitment and 37 weeks gestation between the two groups. We also found no differences in the maternal blood sugars between the two groups. There was, however, a difference in the parity between the two groups (*P* = 0.001). In the fetal parameters measured there were no sex differences in the abdominal subcutaneous tissue, mid-thigh muscle thickness and mid-thigh subcutaneous tissue measurements at 28 and 37 weeks gestation (Table 2). We also found no sex differences in the birth weight (Table 2).

The mean fetal abdominal subcutaneous tissue, mid-thigh muscle thickness and mid-thigh subcutaneous tissue measurements at 28 weeks gestation were 3.4 ± 0.8, 7.7 ± 1.7 and 2.9 ± 0.9 mm, respectively.  Table 3 shows the correlation between maternal parameters, including blood sugars, and fetal body composition at 28 weeks gestation. 

At 28 weeks gestation, maternal age and the maternal one-hour postprandial glucose level both influenced the fetal abdominal subcutaneous tissue measurement independently (*r* ^2^ = 0.095; *P* < 0.001). Maternal age and gestational weight gain both influenced the fetal mid-thigh subcutaneous tissue measurement independently (*r* ^2^ = 0.065; *P* = 0.002). Early pregnancy maternal BMI and fasting glucose levels did not correlate with any of the fetal body composition parameters. 

The mean fetal abdominal subcutaneous tissue measurement, mid-thigh muscle thickness and mid-thigh subcutaneous tissue measurements at 37 weeks gestation were 6.5 ± 1.5, 10.4 ± 2.7, and 5.0 ± 1.6 mm respectively.  Table 4 shows the correlation between maternal parameters, including blood sugars, and fetal body composition at 37 weeks gestation. 

At 37 weeks gestation, the fetal AC measurement correlated with maternal weight and height in early pregnancy and with maternal fasting and one-hour postprandial glucose levels at the time of the normal OGTT (Table 4). To determine which of the variables continued to correlate with the fetal AC measurement, regression analysis was performed incorporating maternal weight, height, and fasting and one-hour postprandial glucose levels. In the resulting regression equation maternal height and fasting and one-hour postprandial glucose levels continued to be predictive (*r* ^2^ = 0.099; *P* < 0.001). The fetal abdominal subcutaneous tissue measurement correlated with all four maternal glucose levels. None of the maternal glucose parameters correlated with the fetal mid-thigh muscle thickness and mid-thigh subcutaneous tissue measurements. 

The fetal AC trajectories from 28 to 37 weeks gestation were influenced by the maternal fasting glucose levels (*F* (1,220) = 4.177, *P* = 0.042). The fetal AC trajectories were not influenced by maternal age, early pregnancy BMI, gestational weight gain and the one-, two- and three-hour postprandial glucose levels. The fetal abdominal subcutaneous tissue trajectories from 28 and 37 weeks gestation were influenced by the maternal three-hour postprandial glucose levels (*F* (1,218) = 4.732, *P* = 0.033). The fetal abdominal subcutaneous tissue trajectories were not influenced by maternal age, early pregnancy BMI, gestational weight gain and the fasting, one and two-hour postprandial glucose levels. The fetal mid-thigh subcutaneous tissue trajectories were not influenced by maternal glucose parameters.

Birth weight correlated with maternal age, height, and parity (*r* = 0.2, 0.2 and 0.2; *P* = 0.024, <0.001 and 0.001 resp.). Birth weight correlated with GWG between the first antenatal visit and 28 weeks gestation (*r* = 0.2; *P* = 0.004) and with GWG between 28 and 37 weeks (*r* = 0.2; *P* = 0.015). The maternal fasting (*r* = 0.1; *P* = 0.04), one-hour postprandial (*r* = 0.2; *P* = 0.008) and 2-hour postprandial (*r* = 0.2; *P* = 0.003) glucose levels all correlated with birth weight. 

To determine which of the above variables continued to correlate with birth weight, regression analysis was performed. The regression equation incorporated maternal age, parity, smoking habit, height, fasting, and one-hour postprandial glucose levels, GWG between booking and 28 weeks, GWG between booking and 28 weeks, and gestational age at delivery. In the resulting regression equation, maternal parity, smoking habit, height, one-hour postprandial glucose level, GWG between booking and 28 weeks, and gestational age at delivery all continued to be predictive of birth weight (*r* ^2^ = 0.306; *P* < 0.001).

## 4. Discussion

In women where GDM was excluded at the start of the third trimester, we found that maternal glycemia correlated with subsequent birth weight. We also found using ultrasound measurement of fetal soft tissues that maternal glycemia influences not only fetal adiposity, but also the distribution of adipose tissue.

We had a high proportion of obese women in our study because one of the indications for an OGTT was a maternal weight >90 kg. Although the women were not representative of our pregnant population, it did, however, allow us to include obese women who did not have GDM. 

In an American study of 220 multiethnic pregnant women with a normal OGTT, women with a BMI ≥25.0 kg/m^2^ had heavier babies (>4.0 kg) than women with a BMI <25.0 kg/m^2^ [16]. Postnatal measurements of neonatal skinfold thickness and total body electrical conductivity estimates of body composition within 72 hours of delivery found that fetal adiposity increased in women who were in the overweight/obese BMI categories. However, it was not possible to comment on the distribution of neonatal adiposity.

In another study of 33 neonates delivered by women with a normal BMI and 39 neonates delivered by overweight/obese women with singleton pregnancies and a normal OGTT, birth weight, and neonatal body composition were compared [17]. Although there was no difference in birth weight between the groups, neonates born to women with a normal pregravid BMI had less fat mass and greater fat-free mass than neonates born to women who were overweight/obese. 

Maternal obesity has been shown to effect glucose metabolism with a loss of the reduction in fasting glucose in early pregnancy and enhancement of peripheral and hepatic insulin resistance [18]. We found that maternal glucose levels, at the time of the normal OGTT, were influenced by early pregnancy BMI. However, we also found that the influence of maternal glucose on fetal body composition was independent of maternal BMI.

An Italian group assessed fetal adiposity at 31 and 37 weeks gestation in fifteen well-controlled insulin-dependent pregnant women and 16 controls with normal glucose [19]. The fasting and postprandial glucose concentrations were higher in the well-controlled diabetic pregnancies compared with the controls. They found no difference in the birth weight and ponderal index between the two groups. However, they found that in the diabetic group fetal abdominal adiposity was increased despite the tight maternal glycemic control.

Another Italian group compared fetal soft-tissue measurements in 228 mothers with normal and abnormal oral glucose challenge tests [20]. The mothers were not obese and did not have GDM. They found that in the women with an abnormal oral glucose challenge test fetal adiposity was increased. This study again suggests that any degree of maternal glycemia represents an altered intrauterine environment for fetal growth even if less than that required for the diagnosis of GDM.

While the numbers in our study are not representative of all pregnant women, it does have strengths. The early pregnancy calculation of BMI and subsequent weight gain was based on accurate measurement throughout pregnancy and was not based on self-reporting [13]. In the HAPO study, BMI was only recorded at the time of the OGTT, which varied between 24 and 32 weeks gestation [8]. We were also able to standardize GWG based on measurements of maternal weight at 28 and 37 weeks gestation allowing us to study the influence of GWG as well as BMI on fetal adiposity [21]. Confining our study to white European women with a singleton pregnancy avoided potential confounding variables such as ethnicity and multiple pregnancies [22]. All women in our study had an early pregnancy ultrasound to confirm gestational age which is a key determinant of estimated fetal weight in utero and of birth weight. The value of measuring fetal adiposity at both 28 and 37 weeks gestation rather than postnatally allowed us to study the relationship between maternal glycemia and intrauterine growth avoiding possible confounding variables such as growth restriction after 37 weeks gestation, neonatal age at measurement and type of infant feeding.

In adults, excess fat deposited abdominally carries higher risk of cardiovascular disease than excess fat deposited in the limbs [23]. Our finding that maternal glycemia independently of GDM influences birth weight, fetal adiposity, and the distribution of fetal adiposity strengthens the possibility that maternal glycemia is a key determinant of intrauterine fetal programming. What implications the increased abdominal adiposity has in later life for the baby's visceral fat levels and risk of metabolic syndrome, however, remains uncertain. Further studies are required to assess whether interventions, such as dietary manipulation, drugs, or exercise, optimize fetal adiposity and birth weight by reducing maternal glycemia even in women who do not have a diagnosis of GDM.

## Figures and Tables

**Table 1:** Characteristics of the study participants, their newborn, and frequency of outcomes (*n* = 231).

Maternal characteristics	Frequency (%)	Mean ± SD	Range
Age (years)		30.6 ± 4.8	19.0–41.0
Parity		0.9 ± 1.0	0–4
Early pregnancy BMI (kg/m^2^)		28.2 ± 6.0	19.1–45.8
Prenatal smoking	14.7		

*Newborn characteristics*			
Gestational age at delivery (weeks)		40.2 ± 1.1	36.6–42.0
Birth weight (kg)		3.7 ± 0.5	2.1–5.0
Birth weight >4.5 kg	3.9		
Male sex	49.8		

*Obstetric outcomes *			
Hypertension			
Gestational hypertension	10.0		
Preeclampsia	3.9		
Induction rate	32.4		
Caesarean delivery	26.5		

BMI: body mass index.

**Table 2:** Mean values for fetal parameters analysed by fetal sex.

	Male fetuses (*n* = 124)	Female fetuses (*n* = 122)	*P* value
*At recruitment *(*28 weeks*)			
Abdominal Circumference (mm)	243.5 (14.5)	243.3 (13.8)	NS
Abdominal subcutaneous tissue (mm)	3.4 (0.7)	3.4 (0.8)	NS
Mid-thigh muscle thickness (mm)	7.7 (1.9)	7.5 (1.6)	NS
Mid-thigh subcutaneous tissue (mm)	2.9 (0.9)	2.9 (0.8)	NS

*At second assessment *(*37 weeks*)			
Abdominal circumference (mm)	331.6 (19.4)	333.0 (16.6)	NS
Abdominal subcutaneous tissue (mm)	6.4 (1.5)	6.6 (1.3)	NS
Mid-thigh muscle thickness (mm)	10.4 (2.8)	10.4 (2.5)	NS
Mid-thigh subcutaneous tissue (mm)	5.2 (1.4)	5.0 (1.4)	NS

*At birth*			
Gestation (weeks)	40.2 (1.2)	40.2 (1.0)	NS
Birth weight (kg)	3.7 (0.5)	3.6 (0.4)	NS

**Table 3:** Correlation between maternal parameters and fetal body composition at 28 weeks gestation (*r*  and *P* values).

Maternal parameter	Fetal AC	Fetal abdominal subcutaneous tissue	Fetal mid-thigh muscle thickness	Fetal mid-thigh subcutaneous tissue
Age (years)	0.1 (NS)	0.3 (<0.001)	−0.1 (NS)	0.2 (0.005)
Parity	0.0 (NS)	0.1 (NS)	0.0 (NS)	0.0 (NS)
*Early pregnancy*				
Weight (kg)	0.0 (NS)	−0.4 (NS)	0.1 (NS)	0.0 (NS)
Height (cm)	0.0 (NS)	0.0 (NS)	0.1 (NS)	0.1 (NS)
BMI (Kg/m^2^)	0.0 (NS)	0.0 (NS)	0.1 (NS)	0.0 (NS)
GWG (1) (kg)	0.1 (NS)	0.0 (NS)	0.1 (NS)	0.2 (0.019)
*OGTT glucose levels *				
fasting	0.0 (NS)	0.1 (NS)	0.0 (NS)	0.0 (NS)
1-hour postprandial	0.1 (NS)	0.2 (0.006)	0.1 (NS)	0.1 (NS)
2-hour postprandial	0.1 (0.026)	0.1 (NS)	0.1 (NS)	0.0 (NS)
3-hour postprandial	0.0 (NS)	0.1 (NS)	0.1 (NS)	0.0 (NS)

AC: abdominal circumference. BMI: body mass index. GWG (1): gestational weight gain per week between the booking visit and 28 weeks gestation. OGTT: oral glucose tolerance test.

**Table 4:** Correlation between maternal parameters and fetal body composition at 37 weeks gestation (*r*  and *P* values).

Maternal parameter	Fetal AC	Fetal abdominal subcutaneous tissue	Fetal mid-thigh muscle thickness	Fetal mid-thigh subcutaneous tissue
Age (years)	0.1 (NS)	0.1 (NS)	−0.1 (NS)	0.0 (NS)
Parity	0.1 (NS)	0.0 (NS)	0.0 (NS)	0.0 (NS)
*Early pregnancy*				
Weight (kg)	0.2 (0.006)	0.0 (NS)	0.1 (NS)	0.1 (NS)
Height (cm)	0.2 (0.007)	−0.1 (NS)	0.1 (NS)	0.1 (NS)
BMI (Kg/m^2^)	0.1 (NS)	0.1 (NS)	0.1 (NS)	0.1 (NS)
GWG (1) (kg)	0.1 (NS)	0.0 (NS)	0.1 (NS)	0.1 (NS)
GWG (2) (kg)	0.1 (NS)	0.1 (NS)	0.0 (NS)	0.2 (0.003)
*OGTT glucose levels *				
fasting	0.2 (0.002)	0.2 (0.006)	0.0 (NS)	0.0 (NS)
1-hour postprandial	0.2 (0.003)	0.2 (0.002)	0.0 (NS)	0.1 (NS)
2-hour postprandial	0.1 (NS)	0.2 (0.014)	0.0 (NS)	0.1 (NS)
3-hour postprandial	0.1 (NS)	0.2 (0.001)	0.0 (NS)	0.0 (NS)

AC: abdominal circumference. BMI: body mass index. GWG (1): gestational weight gain per week between the booking visit and 28 weeks gestation. GWG (2): gestational weight gain per week between 28 and 37 weeks gestation. OGTT: oral glucose tolerance test.
